# Student Burnout and PTSD Symptoms: The Role of Existential Anxiety and Academic Fears on Students during the COVID 19 Pandemic

**DOI:** 10.1155/2022/6979310

**Published:** 2022-01-28

**Authors:** Katarzyna Tomaszek, Agnieszka Muchacka-Cymerman

**Affiliations:** Institute of Psychology, Pedagogical University of Cracow, Poland

## Abstract

It is well known that student burnout is a serious mental health problem, caused by chronic stress related to the educational area. However, in the COVID 19 pandemic, young people have to struggle with additional threats that affect their overall functioning and perception of the world. The main purpose of this study was to investigate the mediating effects of existential anxiety and academic fears on the relationship between academic burnout and posttraumatic stress disorder symptoms. The findings confirmed that academic burnout, existential anxiety, and academic fear were significantly associated with higher posttraumatic symptoms. Existential anxiety and academic fear played a mediating role in the association between academic burnout and posttraumatic stress disorder symptoms. In conclusion, it is considered that student burnout and anxiety indicators are important risk factors for the trauma experienced by students and may increase its symptoms.

## 1. Introduction

Burnout syndrome was initially applied to professional groups, especially those focused on working with people. However, embedding it in an educational context was considered by researchers to be consistent with job burnout. The components of burnout listed in the same way apply to the burnout of adults as well as students. School burnout is an individual characteristic, with a significant impact on education. Experiencing burnout by students consists of exhaustion resulting from the high study demands placed on them by the family or teachers, a cynical and uninvolved approach to study and academic institution, or a sense of inefficiency that are related to negative beliefs about educational competencies or opportunities [1–3]. Nowadays, in an era of constant changes and threats, burnout among students is an increasingly emerging problem affecting people at all levels of education, from primary school to university [4, 5].

Despite numerous definitions that describe people dealing with this mental health problem, there are still differences related to the number of its components and consequences. In particular, little is known about its associations to traumatic stress experienced by a person/student. According to the DMS-V [6] criteria for posttraumatic stress disorder, the exposition or witnessing the traumatic event is a first condition for developing several core PTSD symptoms, e.g., persistent reexperiencing traumatic event (intrusion), negative thoughts and feelings, trauma-related changes in reactivity, and avoidant of people or situations that are connected to trauma (avoidance). An individual who presents signs of posttraumatic stress may also observe exhaustion with all activities, cynicism, and a sense of inefficiency or negative feelings about their abilities. The factors listed above are also burnout components.

### 1.1. Burnout Syndrome and Posttraumatic Stress Disorder (PTSD) Symptoms

The past studies on burnout and PTSD conducted by Mealer et al. [7] revealed that people with PTSD exhibit burnout symptoms. In addition, the coexistence of both symptoms significantly reduces the quality of work but also shifts to personal life satisfaction and world reflections. Research conducted by Rodriguez-Rey et al. [8] combining burnout with PTSD and remedial strategies showed that one-third of respondents who had seen the effects of burnout on PTSD more often used a style focused on dealing with emotions rather than dealing with the problem. The comparison of core symptoms of these two constructs is shown in Table 1. The research results described above indicate a significant relationship between PTSD and burnout; however, little is known about connections between these two constructs among university students. Moreover, the outbreak of the novel coronavirus SARS-CoV-2, responsible for the COVID 19 pandemic, is related to a higher risk of developing mental problems and existential crises, e.g., higher severity of health problems, depression, anxiety, and PTSD among university students [9, 10]. Due to preventive restrictions, distance learning was implemented in schools and universities. Unfortunately, this form of learning negatively impacted the physical and mental health of students at every stage; thus, they are thought to be a vulnerable population. Strict domestic quarantine, social isolation, changes in educational demands, and lack of social and information support were antecedents of higher levels of emotional problems, e.g., fear, generalized anxiety, and acute stress that negatively affected the health-related quality of life of the student population [11, 12].

### 1.2. Educational Burnout and Anxiety Indicators

Students who notice the first signs of burnout also have a high level of anxiety [13]. A meta-analysis conducted by Koutsimini et al. [14] revealed a significant relationship between burnout and anxiety (*r* = .460, SE = .014, 95%CI = .421, .497). In this context, it is worth mentioning the Turnipseed [15] definition, who considered anxiety as a general reaction to threatening situations (state or trait). According to Spielberger [16], some individuals may be more prone to perceive stressful situations as a threat, and the characteristic that is responsible for that tendency is a high anxiety trait.

Stress and anxiety are both emotional responses, but the first construct is typically caused by an external trigger and the second one often includes internal false beliefs about a threat. Additionally, chronic anxiety may be described as persistent, excessive worries that do not disappear even though the stressor has gone. As Cole [17] stated, prolonged anxiety often results in psychological distress negatively affecting an individual's functioning. The stiff pattern of anxiety reactionism uninterrupted and eventually leads to escalation of inappropriate coping behaviors [18]. Anxiety may be associated with many individuals' and social characteristics, e.g., self-requirements and personal false beliefs, teachers' or parents' educational demands and achievement pressure, or the overloading by school/academic work. According to Silvar [19], self-image and anxiety are associated with the development of burnout. Anxiety plays a pivotal role in emotional exhaustion. High students' fear of failure makes them more sensitive to criticism and a chronic feeling of lack of achievement—in a subjective sense. The burned-out individuals are characterized by low impulse control [19], depression, and trait anxiety [20]. A prolonged sense of pressure on achievement may cause anxiety and distress that negatively affect students' effectiveness and mental health [21].

### 1.3. Objectives of the Current Study

Research into academic burnout and posttraumatic stress disorder (PTSD) relationship during coronavirus pandemic COVID 19 among students is limited. Therefore, this study is aimed at identifying the educational and emotional problems predictors of PTSD symptoms during pandemic COVID 19. Examine the role of academic burnout, academic fear, and existential anxiety may have a pivotal role in further intervention based on the past literature; the present study hypothesizes were as follows.

#### 1.3.1. Higher Level of Academic Burnout Will Be Associated with Higher PTSD Symptoms

It is well proven that posttraumatic stress symptoms are connected with higher job related-stress, and some authors have also confirmed a similar connection of these symptoms with burnout among occupations with a high risk of traumatic experience (e.g., firefighters), those that include engaging in dangerous or specialized work abilities and personal responsibilities (e.g., medical services), or those with high expectations to perform socially important or emotionally effort-requiring work (e.g., psychotherapists, correctional psychologists, and teachers) [22, 23]. However, to our knowledge, such connections have not been yet been studied in an educational context.

#### 1.3.2. Higher Existential Anxiety and Academic Fear Will Be Connected with Higher PTSD Symptoms

In the study by Hyland et al. [24], PTSD symptoms described in DSM 5 and ICD-11 were tested in order to check the associations of posttraumatic stress diagnosis with other clinical states such as depression, anxiety, borderline personality disorder, dissociation, destructive behaviors, and suicidal ideation and self-harm. The results indicated that individuals that met the PTSD criteria may be as well diagnosed with another psychiatric disorder. In line with the abovementioned studies, Khodabux et al. [25] found that existential anxiety about the meaning of life is positively connected with lifetime trauma exposure (*r* = .28, *p* < .05) and increased PTSD (*r* = .57, *p* < .001) as well as with reexperiencing (*r* = 0.45, *p* < 0.001), hyperarousal (*r* = .51, *p* < .001), and avoidance (*r* = .53, *p* < .01). Weems et al. [26] found that traumatic stress symptoms that were aroused as a result of exposure to natural disasters (Hurricanes Katrina and/or Gustav) may be predicted by existential (*R*^2^ = .09) and depression symptoms (*R*^2^ = .13). Additionally, disaster exposure moderated the association between subdimensions of existential anxiety and mental health symptoms. It is also confirmed that exposure to childhood trauma is connected with academic problems, emotional and behavioral difficulties, sexually risky behaviors, and substance abuse [27]. Hemiary et al. [28] found that 86.5% of students with PTSD had bad school achievement. Academic fears describe difficulties in a similar area, worries, and concerns felt by young people that are so strong that they make them ineffective in university studying and life.

#### 1.3.3. The Higher the Academic Burnout, the Higher the Anxiety Indicators

Burnout syndrome is defined as a result of chronic academic stress that is not reduced by students because of personal resources depletion and constant overwhelming study demands of the university environment. A large number of prior studies confirmed that such severe stress reaction often triggers anxious states in occupational and educational settings [14]. For example, Mousavi et al. [29] found that burnout explained 25% of the variance in nurses' anxiety. Andriyani et al. [30] stated that students with anxiety feelings are worried in a different number of situations, which in turn increase their stress and burnout levels.

#### 1.3.4. Academic Fear Will Be Indirectly Impacted PTSD Symptoms via Anxiety and Fear Indicators

The studies conducted by Andriyani et al. [30] confirmed the mediation role of anxiety indicators (anxiety as a state and trait) between student burnout and different psychological constructs, such as well-being.

## 2. Materials and Methods

### 2.1. Study Setting and Period

Given the pandemic situation of the outbreak and the purpose of this study, an online anonymous survey on a network platform was designed and published in April 2020. This cross-sectional study was based on a set of psychological tools. The information about the study was distributed among students by several universities' platforms and with the use of popular online messengers and social media. Participants were informed about the possibility of resignation at any time. The sample size requirements were calculated with G^∗^Power-free software [31]. Using a calculator, the sample size for correlation (medium effect with 95% power) was equal to 134 subjects. Due to mediation analysis, the sample size was calculated based on the criteria proposed by Pan et al. [32]. Bootstrapping method to achieve 80% power required 185 subjects.

### 2.2. Study Population

The 199 respondents (169 women, 84.9% of the total sample), with a mean age of 21.92 ± 5.00 years, took part in the study. Most participants studied in the teaching faculties (*N* = 95) and science (*N* = 50). The time of traumatic experience was divided into three groups: (1) from 1 to 5 years ago (*N* = 90), last month to 1 year ago (*N* = 47), and (3) more than 5 years ago (*N* = 62). The traumatic experience mostly was connected with the loss of somebody close to (*N* = 78), work and financial problems (*N* = 44), and sickness or disability (*N* = 34). However, the studies were conducted by the online platform during the COVID19 pandemic; so, all participants might have additionally struggled with the death, disability, or illness threat (see Table 2).

### 2.3. Instruments

The University Students Burnout Scale (USBS) is a 34-item scale with a 4-point Likert scale, based on the SSBS scale originally proposed for secondary school students by Aypay [4]. In the USBS version, the items were adapted to assess academic burnout by matching the content of the questions to university students' situations and problems. It allows measuring students' burnout general level and its seven dimensions. A higher score means a lower academic burnout level. In this study, only the total score was calculated with Cronbach's *α* equal to .92.

Existential Anxiety and Fear Scale—the short version (SNE) by Juros—[33] was used to measure the construct of existential anxiety. The SNE consists of 25 items with the 7-point Likert scale and allows to test the total score of SNE and its three dimensions: (1) fear of guilt and meaninglessness, (2) fear of emptiness and condemnation, and (3) fear of fate and death. In this study, the general level of existential anxiety was used with Cronbach's *α* equal to .94.

The Fears of University Students Scale (FUS) is an experimental 12-item scale with a 7-point Likert scale to assess fears connected with university studying, such as worrying about having lower academic abilities and achievements (i.e., being worse in exams, preparing projects, or oral answers) than other students and concerns regarding making mistakes during tests, exclusion from the team in the situation of group tasks, and fears of public presentation of own projects. The FUS scale reliability measured by Cronbach's *α* was equal to .86.

The Impact Event Scale-Revised (IES-R) created by Weiss and Marmara: in the Polish adaptation of Juczyński and Oginska-Bulik [34], this is a self-report tool to measure symptoms of PTSD that were grouped into three areas: intrusion (Int), hyperarousal (Hyp), and avoidance (Avd). 22 items correspond to the DSM-IV symptoms of PTSD. The Cronbach's *α* was .92 for PTSD total score and ranged between .74 and .89 for its subscales.

### 2.4. Statistical Analysis

IBM SPSS Statistics v.22 with macros presented by Hayes [35] was used for statistical data analyses. Firstly, descriptive statistics (mean and standard deviation, Cronbach's *α*) and Pearsons' analyses were calculated, in order to identify statistically significant relationships between the tested variables. Simple regression analyses were conducted to assess whether the SNE and FUS were associated with USBS, and these, in turn, were linked to greater PTSD. By using the Hayes (2017) macro and multiple mediation models (models 4 and 6), the significance of indirect (mediated) effects of USBS in relation to each of PTSD (separate analyses) through SNE and FUS (mediators) was tested.

### 2.5. Ethics Statement

The study procedure and instruments were approved by the Ethical Commission. Informed consent was obtained from all respondents for participating in the study.

## 3. Results

### 3.1. Pearson's Analysis

Pearson's analysis showed negative and significant associations between academic burnout (USBS) and all posttraumatic stress symptoms (for PTSD *r* = −.26, *p* < .0001, and for its subdimensions ranged between *r* = −.21 and -.22, *p* < .01), as well as with existential anxiety (SNE) (*r* = −.29, *p* < .0001) and academic fear (FUS) (*r* = −.34, *p* < .0001). The results also confirmed significant and positive connections of PTSD with SNE (for PTSD *r* = .36, *p* < .0001 and for its subdimensions *r* between .15 and .38) and with FUS (for PTSD *r* = .24, *p* = .001, for intrusion (Int) *r* = .24, *p* = .001, and for hyperarousal (Hyp) *r* = .21, *p* = .003). The relationship between FUS and avoidance (Avd) was also positive and on the level of tendency (*r* = .14, *p* = .057) (see Table 3).

### 3.2. Simple Mediation Analysis

The findings of the simple mediation analysis showed that there was a significant direct effect of USBS on PTSD (path c), SNE, and FUS (path a). SNE and FUS (mediators) also directly impacted the dependent variable—PTSD (path b). However, mediators reduced USBS impact when tested together (path c′ in models 1 and 2). Similar regularity was found for two PTSD subdimensions—Int and Hyp. SNE and FUS did not mediate the relationship between USBS and the Avd (see Table 4).

Indirect effects were examined with bootstrap method (model 4), and it confirmed the significant mediation role of SNE and FUS on the association between USBS and PTSD, I, and Hyp. The result of mediation analysis for connection between USBS and Avd was insignificant (see Table 5).

### 3.3. Multiple Mediation Analysis

**Figure 1:**
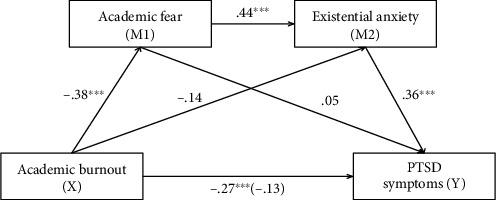
Final results of significant model for two mediators: academic fear and existential anxiety.

Multiple mediation models with two mediators (model 6 in the Hayes macro) were performed in order to test whether USBS impacted indirectly PTSD via both SNE and FUS indicators.  Figure 1 shows the results of model 9, and the total effect (standardized *β* = −.27, SE = .08, *p* = .0006) from USBS to PTSD was at a significant level (step 1). The step 2, direct path from USBS to FUS, (standardized *β* = −.38, SE = .04, *p* < .0001), was significant, and for SNE (standardized *β* = −.14, *p* = .052), it was on the level of tendency. Additionally, the path from the first mediator (FUS) to the second mediator (SNE) was significant (standardized *β* = .44, SE = .19, *p* < .0001) (step 2). The path from the mediator, FUS (standardized *β* = .05, SE = .14, *p* = .515), to independent variable (PTSD) was insignificant and from the second mediator: SNE to PTSD was significant (standardized *β* = .36, SE = .05, *p* < .0001) (step 3). The direct path from USBS to PTSD in step 4 became insignificant (standardized *β* = −.13, SE = .08, *p* = .092). The indirect effect of USBS via two mediators tested simultaneously (FUS and SNE) was also significant (*β* = −.06, SE = .02, CI95%[-.10; -.02]). The parameters of this model were as follows: *R* = .26, *F*_(1,177)_ = 12.24, *p* < .0001 (see Table 5).

## 4. Discussion

The current study investigated the mediation effects of existential anxiety (SNE) and academic fear (FUS) on the relationship between academic burnout (USBS) and PTSD symptoms among university students. Most prior studies on PTSD among students have focused only on early childhood traumatic stress [36] or school bullying/victimization [37]. Additionally, in this study, the relationship between burnout due to studying stress and PTSD symptoms was explored in the university students' sample in the context of the pandemic COVID 19 outbreak. Such a threat increases the concerns of individuals about the meaning of their life, the sense of their being, and the fragility of human life. As Hoffman et al. [38] stated, trauma, by its nature, is existential through its influence on the manner in which the individual experiences the world, their self-understanding, and their sense of place in the world. In our study, past traumatic experiences of students were overlapped by the current threat that generates and activates the mechanisms associated with trauma reenactment. In addition, due to the need to suddenly change the way and conditions of learning (online classes), they had to deal with a number of fears and stresses related to this kind of education and the lack of study resources (libraries) and teachers' support. A direct consequence of such conditions seems to be the increase in existential anxiety about the meaning of life and fears associated with the implementation of academic duties. In turn, such conditions and states are important in the escalation of the burnout symptoms and trauma experience, especially the traumatic reaction that includes existential shattering. According to our results, students were at high risk of experiencing traumatic symptoms when they were burnout and felt existential anxiety and academic fear. Despite the fact that trauma comes in many genres and various degrees of severity, Hoffman et al. [38], our studies shed the light on the educational areas that may increase the posttraumatic stress disorder symptoms and are not directly connected with school victimization. Our study confirmed that the essential part of the healing process for university students with traumatic experiences must include reconstruction of the meaning in life and the sense of self-being, as well as self-belief about the study abilities and the mechanism of coping with university-related stress. When decreasing PTSD among university students, it is vital to include both student burnout and anxiety indicators; otherwise, our actions may be insufficient.

The holistic model of posttraumatic stress disorder treatment assumes strengthening the body and mind so that a person is able to maintain balance [39]. Wilson et al. [40] believe that full recovery from trauma requires respect for life. In treating trauma and traumatic stress, the most important factor is to focus on extracting material to discuss with people, helping to integrate and reduce stress [41]. Wilson et al. [42] believe that in the treatment, the most important thing is to take into account the perception of trauma by the individual who experienced it. In addition, his/her treatment and recovery criteria include four stages: stabilization of symptoms and functioning of the individual in the environment, return to optimal functioning, integration of the individual's experience of trauma, and reduction of the sense of fragmentation. In undertaking the treatment of traumatic stress disorders, it is important to refer to the individuality of its occurrence. On one person, the traumatic situation will not leave a permanent burden, while on another, it will leave a mark in PTSD. This reflects the importance of subjectively assigning meaning to the stimulus from the environment [43]. As Freud noted [44] “…the hysterical symptom disappeared immediately and permanently when we were able to bring to light daily memory of the event by which the symptom was raised along with the accompanying affect, and when the patient described the event as much as possible in detail, the patient translated the effect into words.”

## 5. Limitation of the Study

Several limitations have appeared in the research. The main study limitation is its cross-sectional nature. Therefore, no causal conclusions can be drawn. The second one is gender inequality, as the majority of participants were female. However, according to research, women react more emotionally, especially to stressful situations that have more negative consequences for their functioning [1, 44]. Additionally, the lack of random sampling and the representation of the student population (considering age, gender, income status, etc.) make it difficult to generalize the results. Thirdly, the pertaining results were obtained via self-reported questionnaires; thus, the severity of the academic burnout and fears, anxiety, and PTSD symptoms can be subject to retrospective response bias. The duration of the study (6 months after the outbreak of the coronavirus COVID 19) changed the attitudes and academic stress levels as the students probably have adapted to distance learning. Finally, considering that students voluntarily decided to participate in the survey, a possible selection bias can be also observed. For example, those with more severe mental health problems (educational burnout or PTSD symptoms) would not be willing to attend the psychological research so as not to be forced to reexperience or think about their mental difficulties. Therefore, the prospective verification of the proposed relationship between analyzed psychological constructs based on a larger number of respondents seems valid.

## 6. Conclusion and Recommendations

The most notable finding of this study is that the experience of academic burnout had an indirect effect on PTSD as mediated by existential anxiety and academic fear. Considering the fact that all the abovementioned psychological constructs have a serious negative impact on mental health, in reducing these symptoms among university students, it is recommended to follow a holistic perspective in treatment as well as taking into account the stressors accumulation and existential anxiety of individuals with burnout syndrome and trauma. Based on these results, the policymakers should integrate all efforts aimed to enhance youth mental health during pandemic COVID 19. From the findings, diminishing the PTSD symptoms is strongly related to educational conditions; therefore, teachers and psychological support should be a key part of the didactic process. Our results also underline the universities' need to provide dedicated prophylactic programs to students at higher risk of mental problems. The clinicians' interventions into students with PTSD symptoms should also take into account the strong link between educational and mental health problems to reduce the risk of exacerbation of PTSD and enhance the quality of life of young people. For future researchers, it is important to include studies conducted on children and adolescents to get a more representative picture of the relationship between educational burnout, emotional problems, and PTSD severity. To sum up scientific and clinical recommendations, the strong focus on research concerning students' mental health and educational problems during the ongoing pandemic should be paid.

## Figures and Tables

**Table 1:** PTSD and burnout symptoms–similarities and differences.

PTSD core symptoms (DSM 5, 2013)	Burnout syndrome characteristics
A. Stressor refers to direct or indirect exposure to serious threat (death, injury, sexual violence), and trauma may also occur by witnessing the traumatic situation. B. *Intrusion* (persistently reexperiencing the traumatic event) is defined as such characteristics as unwanted upsetting memories, nightmares, flashbacks, physical reactivity, or emotional distress after coming into contact with a traumatic reminder. C. *Avoidance* of trauma-related stimuli after the traumatic event by trauma-related thoughts or feelings or external reminders related to trauma. D. *Negative alterations* in cognitions and mood that includes negative thoughts or feelings that start to escalate after the trauma experience (e.g., inability to recall key features of the traumatic situation), overall negative attitude (thoughts and affect) towards oneself or the world, decreased interest in activities, the feeling of being isolated, and difficulties in experiencing positive emotions. E. *Alteration in arousal and reactivit*y that began or worsened after the traumatic experience and manifests itself through the following: irritability and aggression, risky or destructive behaviors, hypervigilance, heightened startle reaction, and difficulty in concentrating and sleeping. To meet the B,C, D, and E, it is enough to fulfill at least one symptom. F. Symptoms duration: more than 1 month. G. Distress and functional impairment as consequences of the abovementioned symptoms. H. Symptoms did not meet the criteria of other clinical state or illness and are not connected with the medication action or substance abuse. The DSM V includes two specifications of trauma reactions: dissociative specification with depersonalization and derealization and delayed specification in which full diagnostic criteria have not appeared until at least 6 months after the trauma.	A. Chronic emotional and interpersonal stressors that are directly connected with the occupational or school/university environment. B. Three core symptoms that appear as a prolonged reaction of distress on the aforementioned stressors. (a) *Emotional exhaustion* refers to chronic feelings of fatigue, uneasiness, and being overwhelmed even at the thought of job or school/university) (Salmela-Aro et al., 2009; Maslach, Leiter 2016); anhedonia, persistent negative thoughts and emotions (depressive cognitive style and states, personal worthlessness) (Bianchi, Schonfeld, 2016); difficulties in sleeping due to worrying and ruminating (Pagnin et al., 2014; Vandevala et al., 2017); difficulties in concentration and problem-solving (May et al., 2015); increase in anxiety, nervousness, irritability, violent outbursts, and aggression (Cooper et al., 2017; Oreizi-Esfahani, 2018, Kumar, 2018); and being oversensitive (Kumar, 2018). (b) *Cynical and detached attitude* toward one's job or school/university (depersonalization) (Salmela-Aro et al., 2009; Maslach, Leiter 2016). Loss of interest and engagement in the job or school/academic activities, apathy and boredom, feelings of disappointment, social withdrawal and isolating behaviors, negative attitudes towards coworkers or the school/university social environment, labeling others in derogatory ways (Kumar, 2018), reduction of energy expenditure in working/studying, and finding it meaningless. (c) *Feelings of inadequacy as a professional worker/student*—related to low self-esteem, a decrease in the feeling of personal accomplishment and efficiency in the context of the job or school/academic achievement, feelings of failure and being worse than others, feeling of self-inability, and lack of competence in the field of work or studying (Salmela-Aro et al., 2009; Maslach, Leiter 2016). C. Negative effects on a job or school performance, and achievement, health impairment, and low well-being (Bakker et al., 2018). D. As a consequence dysfunctional behavior occurs: sexually risky behavior, substance abuse, suicidal ideation, technological addiction (to the Internet, mobile).

**Table 2:** Demographic characteristics of participants (*N* = 199).

Characteristics	*N* (%)
Sex	
Female	169 (84.9)
Male	30 (15.1)
Age	
*M* (SD)	21.92 (5.00)
18-20 years	82 (41.2)
21-25 years	105 (52.8)
26-30 years	3 (1.5)
30-48 years	9 (4.5)
Field of study	
Teaching faculties	95 (44.4)
Social sciences	33 (15.4)
Sciences	50 (23.4)
Humanistic science	18 (8.4)
Natural science	2 (0.9)
Lack of data	1 (0.5)
Type of traumatic experience	
Loss of somebody close	78 (36.4)
Work and financial problem	44 (20.6)
Family problems or divorce	26 (12.1)
Sickness or disability	34 (15.9)
Violent event (assault or accident)	14 (6.5)
Other	3 (1.4)
Time from the trauma experience	
From last month to one year	47 (22)
1-5 years	90 (42.1)
>5 years	62 (29)

**Table 3:** Descriptive statistics, reliability, and Pearson's correlation coefficients (*N* = 199).

Variables	*M* (SD)	Alfa	1	2	3	4	5	6	7
1. Intrusion	14.61 (7.94)	.89	—						
2. Hyperarousal	13.55 (6.42)	.81	.84^∗∗∗^	—					
3. Avoidance	13.47 (5.55)	.74	.53^∗∗∗^	.59^∗∗∗^	—				
4. PTSD	41.14 (17.36)	.92	.93^∗∗∗^	.92^∗∗∗^	.78^∗∗∗^	—			
5. Academic burnout^a^	91.24 (15.62)	.92	-.21^∗∗^	-.22^∗∗^	-.21^∗∗^	-.26^∗∗∗^	—		
6. Existential anxiety	98.94 (17.36)	.94	.38^∗∗∗^	.37^∗∗∗^	.15^∗^	.36^∗∗∗^	-.29^∗∗∗^	—	
7. Academic fear	34.91 (10.37)	.86	.24^∗∗^	.21^∗∗^	.14	.24^∗∗^	-.34^∗∗∗^	.49^∗∗∗^	—

^∗∗∗^ *p* < 0.001, ^∗∗^*p* < 0.01, ^∗^*p* < 0.05. ^a^The score in the academic burnout scale is reversed.

**Table 4:** Simple mediation analysis–direct effects.

		*F*	∆*R*^2^	ß	*t*	*p*
Model 1	Independent variable: PTSD total score					
Direct effect	Academic burnout-PTSD total score	12.82^∗∗∗^	.06	-.26	-3.58	<.0001
	Existential anxiety- PTSD total score	27.12^∗∗∗^	.13	.36	5.21	<.0001
	Academic burnout-existential anxiety	17.50^∗∗∗^	.08	-.29	-4.18	<.0001
	Academic burnout and existential anxiety–PTSD total score	21.33^∗∗∗^	.19	ß_USBS_ = −.14	-1.95	.052
				ß_SNE_ = .38	5.34	<.0001
Model 2	Independent variable: PTSD total score					
	Academic fear-PTSD total score	11.32^∗∗^	.05	.24	3.37	.001
Direct effects	Academic burnout-PTSD total score	12.82^∗∗∗^	.06	-.26	-3.58	<.0001
	Academic fear-PTSD total score	11.32^∗∗^	.05	.24	3.37	.001
	Academic burnout-academic fear	25.27^∗∗∗^	.11	-.34	-5.03	<.0001
	Academic burnout and academic fear–PTSD total score	10.34^∗∗∗^	.10	ß_USBS_ = −.18	-2.29	.023
				ß_FUS_ = .21	2.29	.006
Model 3	Independent variable: intrusion					
Direct effects	Academic burnout-intrusion	8.48^∗∗^	.04	-.21	-2.95	.004
	Existential anxiety-intrusion	31.73^∗∗∗^	.14	.38	5.63	<.0001
	Academic burnout-existential anxiety	17.50^∗∗∗^	.08	-.29	-4.18	<.0001
	Academic burnout and existential anxiety-intrusion	22.64^∗∗∗^	.19	ß_USBS_ = −.09	-1.22	.225
				ß_SNE_ = .41	5.94	<.0001
Model 4	Independent variable: intrusion					
Direct effects	Academic burnout-intrusion	8.48^∗∗^	.04	-.21	-2.95	.004
	Academic fear-intrusion	11.58^∗∗^	.05	.24	3.40	.001
	Academic burnout-academic fear	25.27^∗∗∗^	.11	-.34	-5.03	<.0001
	Academic burnout and academic fear-intrusion	9.19^∗∗∗^	.08	ß_USBS_ = −.13	-1.75	.081
				ß_FUS_ = .23	3.08	.002
Model 5	Independent variable: hyperarousal					
Direct effects	Academic burnout-hyperarousal	9.64^∗∗^	.04	-.22	-3.11	.002
	Existential anxiety-hyperarousal	28.94^∗∗∗^	.13	.37	5.38	<.0001
	Academic burnout-existential anxiety	17.50^∗∗∗^	.08	-.29	-4.18	<.0001
	Academic burnout and existential anxiety-hyperarousal	20.25^∗∗∗^	.17	ß_USBS_ = −.10	-1.41	.161
				ß_SNE_ = .39	5.48	<.0001
Model 6	Independent variable: hyperarousal					
Direct effects	Academic burnout-hyperarousal	9.64^∗∗^	.04	-.22	-3.11	.002
	Academic fear-hyperarousal	8.83^∗∗^	.04	.21	2.97	.003
	Academic burnout-academic fear	25.27^∗∗∗^	.11	-.34	-5.03	<.0001
	Academic burnout and academic fear-hyperarousal	9.60^∗∗∗^	.09	ß_USBS_ = −.13	-1.74	.083
				ß_FUS_ = .23	3.05	.003
Model 7	Independent variable: avoidance					
Direct effects	Academic burnout-avoidance	8.16^∗∗^	.04	-.21	-2.86	.005
	Existential anxiety-avoidance	4.19^∗^	.02	.15	2.05	.042
	Academic burnout-existential anxiety	17.50^∗∗∗^	.08	-.29	-4.18	<.0001
	Academic burnout and existential anxiety-avoidance	5.57^∗∗^	.05	ß_USBS_ = −.16	-2.12	.035
				ß_SNE_ = .14	1.80	.073
Model 8	Independent variable: avoidance					
Direct effects	Academic burnout-avoidance	8.16^∗∗^	.04	-.21	-2.86	.005
	Academic fear-avoidance	3.66	.01	.14	1.91	.057
	Academic burnout-academic fear	25.27^∗∗∗^	.11	-.34	-5.03	<.0001
	Academic burnout and academic fear-avoidance	5.48^∗∗^	.05	ß_USBS_ = −.16	-2.00	.047
				ß_FUS_ = .13	1.68	.094

^∗∗∗^ *p* < 0.001, ^∗∗^*p* < 0.01, ^∗^*p* < 0.05. Note: *β*_USBS_: regression coefficient of academic burnout; ß_SNE_: regression coefficient of existential anxiety; ß_FUS_: regression coefficient of academic fear.

**Table 5:** Indirect effects of tested models.

Number of model	Model pathways	Point estimates *β*	SE	95% CI	
				Lower	Upper
1	Academic burnout →existential anxiety→ PTSD total score	-.12	.04	-.20	-.05
2	Academic burnout → academic fear → PTSD total score	-.08	.03	-.15	-.03
3	Academic burnout → existential anxiety → intrusion	-.12	.04	-.22	-.05
4	Academic burnout → academic fear → intrusion	-.08	.03	-.15	-.02
5	Academic burnout → existential anxiety → hyperarousal	-.12	.04	-.20	-.05
6	Academic burnout → academic fear → hyperarousal	-.09	.03	-.16	-.03
7	Academic burnout → existential anxiety → avoidance	-.04	.03	-.10	.00
8	Academic burnout → academic fear → avoidance	-.05	.03	-.11	.01
9	Academic burnout→academic fear→ existential anxiety→PTSD	-.06	.02	-.10	-.02

## Data Availability

All data generated or analyzed during this study are included in this published article.
